# Reviewed article: Davies VF, Azevedo LN, Pinheiro RCL, Baraldi LG. Application of the Food Guide for the Brazilian Population as a training instrument for intersectoral actions: perceptions of professionals in a Brazilian metropolis. Epidemiol Serv Saude. 2025:34;e20240397.

**DOI:** 10.1590/S2237-96222025v34e20240397.a

**Published:** 2025-05-23

**Authors:** Amanda Nathale Soares

**Table:** 

Rating	Excellent	Good	Average	Below average	Poor
Interest			✓		
Quality			✓		
Originality		✓			
Overall			✓		

**Table:** 

Questionnaire	Yes	No	Not applicable
Does the manuscript contain new and significant information to justify publication?	✓		
Does the Abstract (Summary) clearly and accurately describe the content of the article?	✓		
Is the problem significant and concisely stated?	✓		
Are the methods described comprehensively?	✓		
Are the interpretations and conclusions justified by the results?	✓		
Is adequate reference made to other work in the field?	✓		

## Manuscript Structure:

Length of article is: Too short

Number of tables is: Adequate

Number of figures is: Adequate

Please state any conflict(s) of interest that you have in relation to the review of this paper (state “none” if this is not applicable): None

## Comments

O artigo propõe-se a apresentar a análise de uma intervenção educativa, fundamentada no Guia AlimentarBrasileiro e realizada junto a profissionais da Saúde e da Educação que atuam com a população infantil.Considero muito importante e necessária a publicação de estudos que deem visibilidade a práticasformativas realizadas com profissionais que atuam em diferentes contextos que possuem interface com acomunidade/população, a exemplo da Saúde e da Educação. Por isso, considero interessante a proposta doartigo, sobretudo por se tratar de uma intervenção realizada, conjuntamente, com profissionais de ambos ossetores. Entendo que a experiência da intervenção educativa e também o conteúdo das entrevistasrealizadas com profissionais participantes possuem aspectos muito importantes e pertinentes de seremcompartilhados, mas, para que haja maior potencial de publicação, considero ser necessária uma amplareformulação do texto, a partir de algumas questões principais:

• É necessário um detalhamento do modo como a intervenção educativa foi realizada. Para além dasinformações de organização da intervenção, como carga horária, formato e temáticas abordadas, como sederam os processos de ensino-aprendizagem, considerando que a proposta “incentivou a construção crítica-reflexiva entre os participantes e facilitadores, ampliando a compreensão do conteúdo do Guia e suasestratégias de aplicação na prevenção da obesidade infantil, além de fomentar a reflexão sobre as práticasprofissionais no contexto intersetorial”? Que método e estratégias pedagógicos orientaram odesenvolvimento das oficinas? Entendo que, por se tratar de uma análise de intervenção educativa, éimportante compartilhar as apostas pedagógicas que a orientaram.

• É necessário maior aprofundamento das questões apresentadas no âmbito dos resultados e da discussão:as falas dispostas no Quadro 2 apresentam conteúdos que permitem e exigem maior aprofundamento dasquestões levantadas sobre a intervenção educativa. As sínteses sobre as falas, apresentadas na seção“resultados”, explicitam, de modo muito superficial, os conteúdos das entrevistas. Sugiro que trechos dasfalas dos participantes sejam apresentados na seção “resultados”, a partir dos quais sejam melhorexploradas e discutidas as questões que apontam, analiticamente, os desdobramentos da intervençãoeducativa.

• É necessária uma revisão ortográfica de todo o artigo.

• Sugiro, ainda, sintetizar o título do artigo, para facilitar a comunicação com o leitor, e substituir o verbo“avaliar uma intervenção educativa” por “analisar”, considerando que o termo “avaliação” pode remeter areferenciais e estratégias específicos, que não foram objeto metodológico no âmbito do estudo realizado.

